# Isolation, identification, and pathogenicity of porcine epidemic diarrhea virus

**DOI:** 10.3389/fmicb.2023.1273589

**Published:** 2023-10-13

**Authors:** Yingshuo Sun, Ting Gong, Dongdong Wu, Yongzhi Feng, Qi Gao, Jiabao Xing, Xiaoyu Zheng, Zebu Song, Xing Liu, Xiongnan Chen, Yankuo Sun, Guihong Zhang, Lang Gong

**Affiliations:** ^1^Guangdong Provincial Key Laboratory of Zoonosis Prevention and Control, College of Veterinary Medicine, South China Agricultural University, Guangzhou, China; ^2^Key Laboratory of Animal Vaccine Development, Ministry of Agriculture and Rural Affairs, Guangzhou, China; ^3^Zhaoqing Branch Center of Guangdong Laboratory for Lingnan Modern Agricultural Science and Technology, Zhaoqing, China

**Keywords:** porcine epidemic diarrhea virus, mutation, nsp1, identification, pathogenicity

## Abstract

Porcine epidemic diarrhea (PED) is an enterophilic infectious disease caused by the porcine epidemic diarrhea virus (PEDV), which can lead to dehydration-like diarrhea in piglets with a mortality rate of up to 100%, causing huge economic losses to the global pig industry. In this study, we isolated two PEDV strains, FS202201 and JY202201, from diarrheal samples collected from two new PED outbreak farms in 2022. We performed phylogenetic analysis of the S gene and whole gene sequence. The effects of the different mutations on viral pathogenicity were investigated using piglet challenge experiments. The results showed that both strains belong to the G2c subtype, a widely prevalent virulent strain. Compared with FS202201, JY202201 harbored substitution and deletion mutations in nsp1. Both FS202201 and JY202201 infected piglets showed severe diarrhea and significant intestinal tissue lesions at an infection dose of 10^4^ TCID_50_/mL, with a mortality rate of 50%; however, JY202201 required an additional day to reach mortality stabilization. An infection dose of 10^3^ TCID_50_/mL reduced diarrhea and intestinal tissue lesions in piglets, with mortality rates of the two strains at 16.7% and 0%, respectively. In addition, PEDV was detected in the heart, liver, spleen, lungs, kidneys, mesenteric lymph nodes, stomach, large intestine, duodenum, jejunum, and ileum, with the highest levels in the intestinal tissues. In conclusion, this study enriches the epidemiology of PEDV and provides a theoretical basis for the study of its pathogenic mechanism and prevention through virus isolation, identification, and pathogenicity research on newly identified PED in the main transmission hub area of PEDV in China (Guangdong).

## 1. Introduction

Porcine epidemic diarrhea (PED), caused by porcine epidemic diarrhea virus (PEDV), can occur in all age groups; however, piglets are the main group affected because of their incomplete immune system, manifesting as acute diarrhea, vomiting, and dehydration, with a mortality rate of up to 100%. PEDV was first isolated in the UK in 1971 and spread rapidly throughout Europe (Pensaert and de Bouck, 1978). PEDV was first identified in Asia in 1982, and has since been reported in various Asian countries (Takahashi et al., 1983). Although it was widely prevalent, its low mortality rate meant it did not attract much attention. In 2010, a highly pathogenic mutant strain with a 100% mortality rate emerged in China, rapidly spreading to many Asian countries (Chen et al., 2011; Wang et al., 2016). This highly virulent variant spread to the United States in 2013, causing a total loss of $400 million in the pig industry in 31 states over a short period (Stevenson et al., 2013; Lowe et al., 2014; Jung and Saif, 2015). A similar strain emerged in Germany in 2014, followed by reports of PED in France, Belgium, Italy, Austria, and Spain among the European countries (Hanke et al., 2017). Therefore, PEDV is regarded as one of the most devastating pig viruses, causing significant economic losses to the global pig industry. Presently, PEDV has become a very important pig pathogen in China, second only to African swine fever virus and porcine reproductive and respiratory syndrome virus (Wang et al., 2019). Within China, the Guangdong and Henan provinces have been identified as major hubs for PEDV transmission, with the pig trade playing a crucial role (He et al., 2022).

PEDV, a member of the genus *Alphacoronavirus*, is an enveloped, positive-sense single-stranded RNA virus. Its genome size is approximately 28 kb, and it encodes four structural proteins (S, E, M, and N), 16 non-structural proteins (nsp1–nsp16), and an auxiliary protein ORF3 (Lin et al., 2022). The S protein is a major determinant of virulence and mediates viral entry into host cells (Li, 2016). Mutations or insertions in the S1 region (1–789 amino acids) affect virulence (Suzuki et al., 2018). Therefore, the S gene is commonly used for genetic and evolutionary analyses to distinguish between PEDV strains. Among the non-structural proteins, nsp1, nsp3, nsp5, nsp7, nsp14, nsp15, and nsp16 can serve as interferon antagonists and regulate the natural immune response, with nsp1 having the most significant effect on the host’s innate immune response (Zhang et al., 2016). Further, nsp1 is considered an important virulence determinant and target for vaccine development as it can interfere with host gene expression and stimulate antiviral responses, thereby blunting the host’s innate immune response to coronavirus pathogens (Hu et al., 2021). It has been shown that nsp1 mutations affect the virulence of PEDV (Niu et al., 2022).

To determine the biological characteristics and strain evolution of new PED outbreaks under the current strict epidemic control situation, we isolated two PEDV strains from the main area of PED transmission (Guangdong), namely FS202201 and JY202201. The identification and genomic sequence analysis of these two virulent strains were performed. Through piglet challenge experiments, we evaluated the impact of different mutations on pathogenicity and found that strain JY202201 had lower pathogenicity than strain FS202201. This study is expected to enrich the background knowledge of the current PEDV-prevalent strains and provide a theoretical basis for the study of the pathogenic mechanism of PEDV.

## 2. Materials and methods

### 2.1. Sample collection and cells

In 2022, a commercial farm in Guangdong Province experienced a disease outbreak in pig farms in the cities of Foshan and Jieyang. The main manifestations were depression, anorexia, vomiting, and dehydration-like diarrhea. Dying piglets with typical symptoms were euthanized (150 mg/kg pentobarbital sodium was injected into the ear vein) and immediately autopsied. We collected fresh, lesional intestinal tissue from both farms. Vero cells were cultured in Dulbecco’s modified Eagle’s medium (DMEM) (Gibco, Shanghai, China) supplemented with 10% fetal bovine serum (Gibco, Guangzhou, China).

### 2.2. Virus isolation and identification

The intestinal tract was incised longitudinally and the long villi and intestinal contents were scraped with a cell scraper. An equal amount of DMEM was added and mixed for freeze-thaw cycles. The samples were centrifuged at 4°C at 8,000 rpm/min for 10 min. The supernatant was decontaminated using a 0.22-μm filter and dispensed for virus isolation. Vero cells were used for amplification and isolation of viruses. The inoculum was maintained for 3 days in DMEM containing 7 μg/mL trypsin (Gibco, Guangzhou, China). Each virus generation was assayed for viral proliferation and cytopathic effects. After stable passages, the viruses were purified, and their morphology was identified using a transmission electron microscope (FEI, Brno, Czechia).

### 2.3. Reverse transcription PCR (RT-PCR) and RT-qPCR

Viral RNA was extracted from the virus supernatant using the Fastagen kit and reverse-transcribed into cDNA using iScript Reverse transcriptase (Thermo Fisher Scientific, Guangzhou, China). The PEDV-N fragment was amplified using forward and reverse primers targeting the N gene (forward, 5′-TGGCTTCTGTCAGCTTCCAGGATC-3′; reverse, 5′-ATTTC CTGTATCGAAGATCTCGTT-3′). The products were analyzed by electrophoresis on a 1% agarose gel. According to the ChamQ Universal SYBR qPCR Master Mix (Vazyme, Q711-02, Nanjing, China) instructions, virus RNA content was determined using forward and reverse primers targeting the N gene (forward, 5′-CACCTCCTACTTCACGTGCA-3′; reverse, 5′-AGCTCACCGACCTGGTTAT-3′).

### 2.4. Immunofluorescence assay

After 24 h of inoculation, Vero cells were fixed with 4% paraformaldehyde for 15 min at room temperature and permeabilized with 0.3% Triton-X100 for 15 min at room temperature. The infected cells were treated with PEDV-N specific monoclonal antibody as the primary antibody for 2 h at 37°C, CoraLite488 goat anti-mouse as the fluorescent secondary antibody for 45 min at 37°C, and DAPI for the nuclei for 5 min at room temperature. Each step was followed by washing thrice with phosphate buffered saline. Finally, the cells were observed under a fluorescence microscope (Leica, Wetzlar, Germany).

### 2.5. Viral growth kinetics

The growth kinetics of endemic strains GDS01, FS202201 and JY202201 were measured by the 50% tissue culture infective dose (TCID_50_). The virulent strains were inoculated into monolayers of Vero cells and the supernatants were collected at 0, 3, 6, 12, 24, and 36 h and stored at −80°C. Virus titers were assayed using a 96-well plate by diluting the virus at serial tenfold dilutions and repeating three times. The cytopathic effects were identified and TCID_50_ was calculated according to the Reed-Muench formula (Yu et al., 2018).

### 2.6. Next-generation sequencing (NGS)

RNA was extracted as described previously. Host ribosomal RNA (human/mouse/rat) were removed using a Ribo-MagOff rRNA Depletion Kit (Vazyme, Nanjing, China) during the library construction, and all of the 150 bp pair-end sequencing libraries were constructed using a VAHTS^®^ Universal V8 RNA-seq Library Prep Kit for Illumina (Vazyme, Nanjing, China). The quality and quantity of the library alignment were further assessed using a Qubit 4.0 fluorometer (Invitrogen, Carlsbad, CA, USA) and an Agilent 2100 Bioanalyzer (Agilent Technologies, Santa Clara, CA, USA), respectively, before sequencing. Finally, the qualified libraries were sequenced using a NovaSeq 6000 sequencer (Illumina, San Diego, CA, USA). Trimmomatic was used to trim the short-segment adaptation sequences and remove low-quality reads (quality scores < 20). We used MEGAHIT to assemble the reads and generate the entire contigs. Finally, we employed SAMtools and iVar to map and obtain the consensus genome of PEDV, with fine scrutiny of coverage information. The complete gene sequence has been submitted to GenBank (OR418363 and OR418364).

### 2.7. Genomic analysis

First, we retrieved all available S genes and complete genome reference sequences for PEDV from the National Center for Biotechnology Information nucleotide database (GenBank) and downloaded the entire database. They were aligned using MAFFT v7. Sequences with homology higher than 95% were removed using CD-hit. Maximum likelihood phylogenies were estimated using IQ-TREE v.1.6.5, with 1,000 bootstrapped replicates to generate the consensus phylogenies of the S gene and complete genome. The results were visualized using Figtree v1.4.4^1^ and ggtree in R. In addition, amino acid translation and sequence alignment were performed on the viral sequences using the EditSeq and MegAlign programs (DNAStar7.1) (Zhu et al., 2022), and the results were further visualized using Jalview.

### 2.8. Pathogenicity assessment of isolated PEDV strains FS202201 and JY202201 in piglets

#### 2.8.1. Animal experiment design

Thirty 3-day-old piglets that tested negative for ASFV, CSFV, PRRSV, PEDV, PDCoV, TEGV, PRoV, and JEV were randomly grouped. Among them, six were in the negative control group. Twelve in the FS202201 infection group, and six each in the 10^4^ TCID_50_/mL and 10^3^ TCID_50_/mL infection doses groups. Twelve in the JY202201 infection group, and six each in the 10^4^ TCID_50_/mL and 10^3^ TCID_50_/mL infection doses groups. The first day was adaptive cultivation and the second day was an artificial oral challenge. The negative control group received DMEM orally, using an identical method. Each piglet group was kept apart and nurtured in separate rooms.

#### 2.8.2. Clinical symptoms

The duration of the experiment was 14 days, and the clinical symptoms of the piglets were observed every day after the virus infection. Diarrhea was scored according to the scoring criteria listed in Table 1. We also recorded daily changes in weight, body temperature, and survival rate of the piglets in each group.

**TABLE 1:** Diarrhea scoring criteria.

Clinical symptoms	Scoring criteria	Score
Diarrhea level	Normal feces	0
	Soft feces	1
	Loose feces	2
	Watery feces	3

#### 2.8.3. Histopathology

Dying piglets were euthanized and immediately autopsied to observe general changes and to collect tissues. The intestinal tissues (jejunum and ileum) were fixed in 10% formalin and removed for water washing, dehydration, and transparency, before immersion waxing and embedding. The tissue was cut into five-micron-thick sections using a microtome, dewaxed, and stained with hematoxylin and eosin (HE) (Yu et al., 2019). Pathological sections were observed under a light microscope (Leica, Wetzlar, Germany).

#### 2.8.4. Detection of viral loads

Anal swabs were collected daily from the piglets to detect the virus content *in vivo* using RT-qPCR. Equal amounts (1 g) of tissue samples (heart, liver, spleen, lung, kidney, lymph nodes, stomach, large intestine, duodenum, jejunum, and ileum) were placed in 5 mL of phosphate buffered saline, freeze-thawed twice, and ground using a grinder. The samples were then centrifuged at 15,000 × *g* at 4°C for 10 min. RT-qPCR was performed to determine the viral RNA copy number in the supernatant. Each piglet was subjected to these tests.

### 2.9. Statistical analysis

All data are expressed as mean ± standard deviation and were analyzed by the Student’s *t* test. Statistical significance is indicated by asterisks (*, *p* < 0.05; **, *p* < 0.01; ***, *p* < 0.001; ****, *p* < 0.0001). All the statistical analyses were performed using the program in GraphPad Prism 7.0.

## 3. Results

### 3.1. Isolation and identification of viruses

**FIGURE 1:**
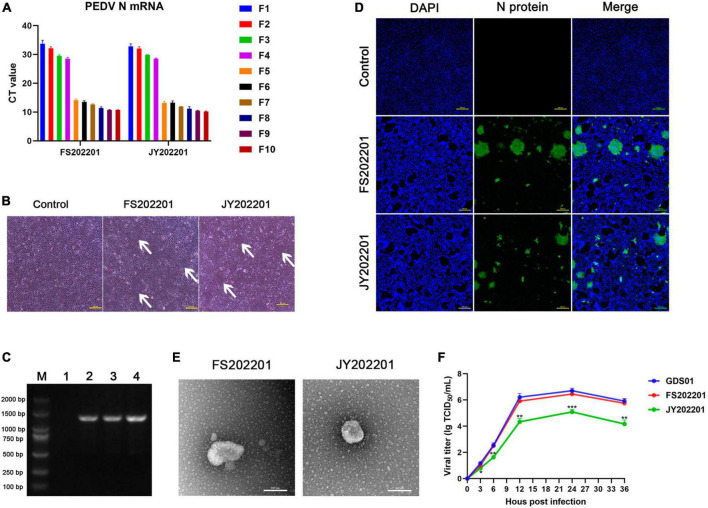
Identification of isolated porcine epidemic diarrhea virus (PEDV) strains FS202201 and JY202201. **(A)** Detection of PEDV-N gene expression levels in virus supernatants of generation 1–10 using RT-qPCR. **(B)** Vero cells infected with the strains FS202201 and JY202201 observed with a light microscope. White arrows indicate the syncytium area. **(C)** PCR amplification products of the virus supernatant; (M) A 2,000 bp marker, (1) Dulbecco’s modified Eagle’s medium containing trypsin as negative control; (2) Strain GDS01; (3) Strain FS202201; (4) Strain JY202201. **(D)** Vero cells infected with the strains FS202201 and JY202201 were detected using immunofluorescence assay. **(E)** Purification of PEDV virus particles obtained from the supernatant of the strains FS202201 and JY202201, observed by transmission electron microscope. **(F)** The growth kinetics of the strains GDS01, FS202201 and JY202201 in Vero cells. FS202201 and JY202201 were statistically analyzed against GDS01, respectively (*, *p* < 0.05; **, *p* < 0.01; ***, *p* < 0.001).

To isolate the FS202201 and JY202201 strains, the processed intestinal contents were passaged in Vero cells. In the first 10 generations, RT-qPCR assays showed the mRNA level of PEDV-N gene increased in the viral supernatant from the fifth generation onward (Figure 1A). Cytopathic effects were observed in Vero cells inoculated with strains FS202201 and JY202201, both showing syncytium formation (Figure 1B). RT-PCR detection of the PEDV-N gene was positive with a size of 1,300 bp (Figure 1C). Vero cells inoculated with FS202201 and JY202201 strains were further examined by immunofluorescence assay. The PEDV-N protein-specific green fluorescence and the syncytia were observed. This demonstrated that Vero cells were infected by PEDV, consistent with the previous results (Figure 1D). The purified virus particles were round with coronal protuberances, as observed by transmission electron microscopy, and their diameters were approximately 80–120 nm (Figure 1E). The *in vitro* results of viral growth kinetics showed that there was no significant difference between the FS202201 strain and Guangdong endemic strain GDS01, while JY202201 was weakly replicating compared to strain GDS01 (Figure 1F). In summary, we successfully isolated PEDV.

### 3.2. Phylogenetic tree of strains FS202201 and JY202201

**FIGURE 2:**
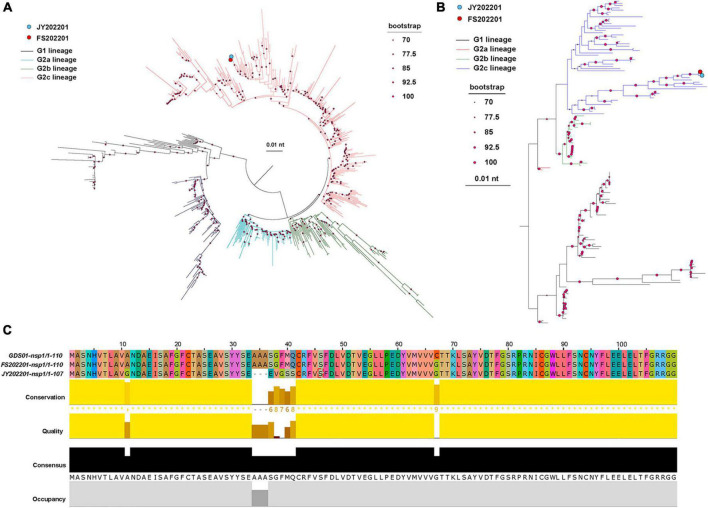
Phylogenetic analysis and amino acid comparison of PEDV. **(A)** S gene phylogenetic tree. The blue dot represents strain JY202201, and the red dot represents strain FS202201. The evolutionary branches of different genomes are colored as follows: G1 (black), G2a (blue), G2b (green), and G2c (red). The size of the dots at the nodes represents the bootstrap values. The scale bar represents the nucleotide substitution at each site. **(B)** Whole genomic phylogenetic tree. The evolutionary branches of different genomes are colored as follows: G1 (black), G2a (red), G2b (green), and G2c (blue). **(C)** nsp1 amino acid sequence alignment of strains GDS01, FS202201, and JY202201. Colors represent homologous amino acids. Bars represent each amino acid’s conservation, quantitative measurement, and frequency of occurrence.

We sequenced the two isolates to determine whether the outbreaks of PED on the two farms were caused by different strains. The results showed that we isolated two PEDV strains, FS202201 and JY202201. Phylogenetic trees were constructed based on the S gene and the whole genome of the PEDV virus, which indicated that strains FS202201 and JY202201 belong to the G2c subtype. The nucleotide sequences of the strains FS202201 and JY202201 shared 99.9% similarity. Both strains shared a single cluster, indicating a relatively homogeneous evolutionary relationship of PEDV contemporarily circulating in southern China (Figures 2A, B).

### 3.3. Genomic sequence analysis

Since the differences in the sequences of the two strains are centered in nsp1. We compared the nsp1 amino acid sequences of strains FS202201, JY202201, and Guangdong endemic strain GDS01, and the results showed that strain JY202201 had deletion mutations (A34–A36) and substitution mutations (A37–A41) in nsp1 (Figure 2C).

### 3.4. Pathogenicity of PEDV strains FS202201 and JY202201

#### 3.4.1. Clinical symptoms

**FIGURE 3:**
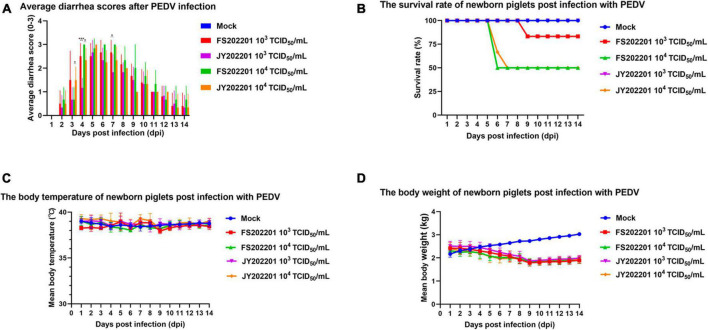
Clinical symptoms of inoculation with PEDV strains FS202201 and JY202201. **(A)** Bar graph of piglet diarrhea scores (*, *p* < 0.05; ***, *p* < 0.001). **(B)** Survival rate curve of piglets. **(C)** Graph of body temperature change in piglets. **(D)** Graph of weight change in piglets.

To verify the pathogenicity of the FS202201 and JY202201 strains, piglet attack experiments were conducted. The results showed that the control group did not develop symptoms of diarrhea and mental depression. Diarrhea was generally more severe in the FS202201 group than in the JY202201 group for the same infection titer (Figure 3A). The FS202201 group, with a dose of 10^3^ TCID_50_/mL, had a death at 9 dpi, whereas the JY202201 group had no deaths. With 10^4^ TCID_50_/mL, the mortality rate reached 50% at 6 dpi in the FS202201 group but at 7 dpi in the JY202201 group (Figure 3B). Compared with the control group, the experimental group showed no significant change in body temperature (Figure 3C). While body weights showed a decreasing trend from 5 dpi (Figure 3D). After 9 dpi, the piglets no longer died and clinical symptoms improved.

#### 3.4.2. Gross lesions

**FIGURE 4:**
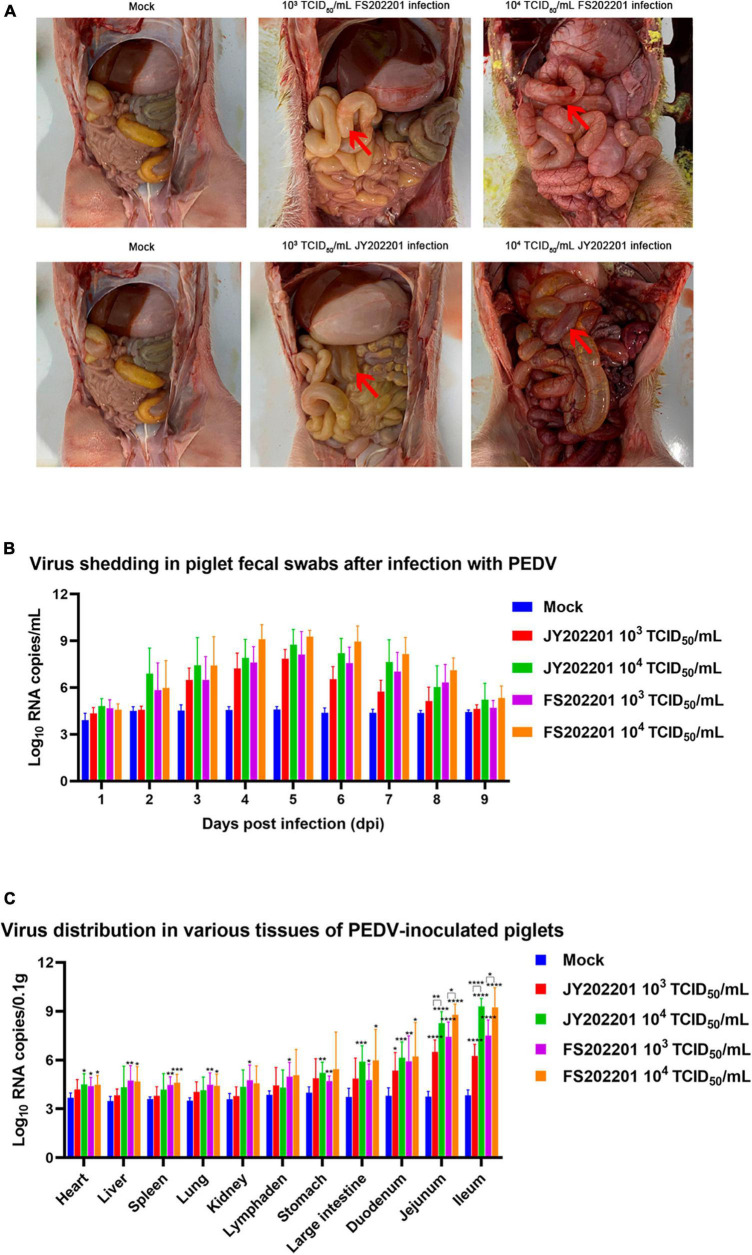
The gross anatomy and body viral load of piglets inoculated with PEDV strains FS202201 and JY202201. **(A)** The gross anatomy and organ lesions of piglets in the control group and experimental group (with obvious symptoms). Red arrows indicate the pathological intestinal tract. **(B)** PEDV viral load in anal swabs. **(C)** PEDV viral load in each organ (*, *p* < 0.05; **, *p* < 0.01; ***, *p* < 0.001; ****, *p* < 0.0001).

The autopsy results showed no pathological changes in the control group. As shown by the red arrows, in the FS202201 and JY202201 groups, a large amount of yellow liquid had accumulated in the small intestine, with transparent and thin intestinal walls and inflated intestines. The intestinal wall was more transparent in the 10^4^ TCID_50_/mL group. In all groups, no lesions were observed in other organs (Figure 4A).

#### 3.4.3. Viral load

We collected anal swabs daily for 9 days after the virus infection, and RT-qPCR was used to detect the changes in virus content within the piglets. Although there were no statistical differences between groups, the FS202201 group generally had higher viral RNA copy numbers. Piglets in both the 10^4^ TCID_50_/mL and 10^3^ TCID_50_/mL groups showed peak viral shedding at 5 dpi (Figure 4B).

The viral load in different tissues of piglets was measured using RT-qPCR. Compared to controls, viral RNA copy numbers were elevated in the heart, liver, spleen, lungs, kidneys, mesenteric lymph nodes, stomach, colon, duodenum, jejunum, and ileum, with the most significant elevations in intestinal tissues (duodenum, jejunum, and ileum). The virus content in various organs increased with the increase in virus titer, which was most evident in intestinal tissues (jejunum and ileum) (Figure 4C).

#### 3.4.4. Histopathology

**FIGURE 5:**
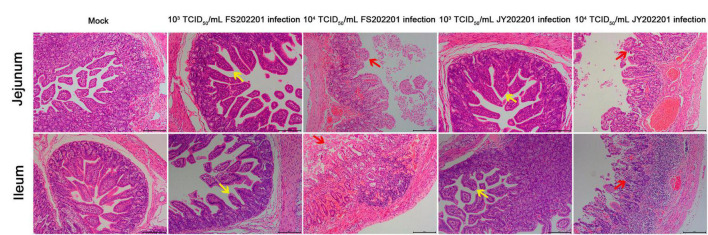
Histopathological lesions in the jejunum, and ileum of piglets inoculated with PEDV strains FS202201 and JY202201. Yellow arrows indicate the villi atrophied. Red arrows indicate the villi severely atrophied and detached.

To further determine the pathogenicity of FS202201 and JY202201 in the intestine, we collected jejunal and ileal tissues to analyze histological changes. The results showed that compared with the control group, the jejunal and ileal villi were severely atrophied or detached in the 10^4^ TCID_50_/mL group, as indicated by the red arrows. We also observed necrotic shedding of intestinal epithelial cells, and severe necrosis and structural disintegration of the mucosal layer. The lamina propria was severely congested, and numerous necrotic exfoliated intestinal tissues were observed in the intestinal cavity. In the JY202201 strain 10^4^ TCID_50_/mL group, the inflammatory cell infiltration in the lamina propria was predominantly lymphocytes, with a few plasma cells and monocyte-macrophages seen. In the 10^3^ TCID_50_/mL group, the jejunal and ileal villi were atrophied, as indicated by the yellow arrow. The FS202201 strain caused generally more severe tissue lesions than the JY202201 strain, which is consistent with the previous findings (Figure 5).

## 4. Discussion

After a highly pathogenic PEDV strain emerged in China in 2010, this mutant strain rapidly spread, causing significant economic losses to the global pig industry (Li et al., 2012). In China, PED has occurred in almost all provinces, and PEDV has been the dominant virus in the past decade (Tan et al., 2020; Zhang et al., 2021; Li et al., 2022a). Most RNA viruses, which undergo a high rate of errors during their genomic replication, have a high mutation rate (Niu et al., 2021). PEDV, as a major pandemic RNA virus, has strains that continue to mutate along with time, resulting in changes in virulence and pathogenicity (Chen et al., 2019; Zhang et al., 2022). We collected new PEDV-positive cases from Guangdong Province, China, and successfully isolated two PEDV strains: FS202201 and JY202201. Some studies have shown that China has the most diverse PEDV populations, and Guangdong Province and Henan Province are the primary hubs in China (He et al., 2022; Zhang et al., 2023). Therefore, the isolates from this experiment are of epidemiological significance. We further investigated the genetic evolution of FS202201 and JY202201, and their pathogenicity in piglets.

Studies have shown that the S protein plays an important role in the process of PEDV cell entry, assembly, and induction of neutralizing antibodies against PEDV (Lin et al., 2022). Mutations in the S protein amino acid sequence may affect the virulence and pathogenicity of PEDV (Liu et al., 2022). Therefore, the S gene is commonly used to evaluate genetic diversity and evolution. In this study, the phylogenetic trees of strains FS202201 and JY202201 were analyzed based on the S gene and the whole genome, and both strains belonged to G2c genotype. It was reported that G2c was the most prevalent strain in China from 2017 to 2021 (Li et al., 2022b). Many new G2b vaccines do not provide sufficient protection against G2c PEDV strains in China, and successive passages can lead to the attenuation of G2c genotype strains (Ge et al., 2022). Therefore, FS202201 and JY202201 contribute to the development of effective vaccines.

Strains FS202201 and JY202201 were obtained from different cities of the same commercial pig farm, and the pigs were transported between farms. Therefore, the whole-gene base homology between the two strains was 99.9%. Notably, the mutation site in strain JY202201 was concentrated in the nsp1 compared to strain FS202201. The nsp1 in strain JY202201 had substitution and deletion mutations that resulted in amino acid changes. Coronavirus nsp1 is involved in shutting down the host translation system and is an important virulence determinant (Hu et al., 2021; Nakagawa and Makino, 2021). It has been shown that PEDV nsp1 can repress host gene expression and the nsp1 N93A and N95A mutations can attenuate PEDV (Shen et al., 2018; Niu et al., 2022). Therefore, the natural transmission of virulent strains leading to mutations is of great interest for research.

In the pathogenicity assay, compared with strain FS202201, strain JY202201 required one more day to stabilize the mortality rate in the 10^4^ TCID_50_/mL group and had a lower mortality rate in the 10^3^ TCID_50_/mL group. The clinical signs and pathological changes were relatively mild in piglets in the JY202201 group. Although increased PEDV replication was detected in all organs, increased viral replication was most pronounced in intestinal tissues (duodenum, jejunum, and ileum). Combined with the pathological changes, the results indicate that the main target organ of FS202201 and JY202201 strains is the intestine, which is consistent with the results of previous studies (Jung et al., 2020). The *in vitro* replication kinetics results showed that JY202201 strain also exhibited reduced virulence *in vitro*. Taken together, we hypothesized that the reduced virulence of strain JY202201 was mainly caused by the nsp1 mutation; however, this hypothesis requires further verification.

In the present study, intestinal disease samples were collected from newly diagnosed PED pig farms. Two PEDV strains, FS202201 and JY202201, were isolated. After identification, both strains belonged to the G2c subtype. The pathogenicity of the two isolates was verified using piglet challenge experiments. We found that the pathogenicity of JY202201 was relatively diminished, which may be due to the concentration of its mutation in nsp1. Through the above studies, we conducted a preliminary discussion on whether nsp1 mutation affects the pathogenicity of PEDV, and we will be conducted on the pathogenesis in the future studies. In summary, this study contributes to the current knowledge of PEDV epidemiology and provides a foundation for understanding its genetic variation and pathogenicity.

## Data availability statement

The original contributions presented in this study are included in this article/supplementary material, further inquiries can be directed to the corresponding author.

## Ethics statement

The animal study was approved by the Experimental Animal Ethics Committee of South China Agricultural University. The study was conducted in accordance with the local legislation and institutional requirements.

## Author contributions

YS: Data curation, Writing—original draft. TG: Validation and Data curation. DW: Validation, Writing—review and editing. YF: Validation, Writing—review and editing. QG: Data curation, Writing—review and editing. JX: Software, Writing—review and editing. XZ: Validation, Writing—review and editing. ZS: Investigation, Writing—review and editing. XL: Investigation, Writing—review and editing. XC: Investigation, Writing—review and editing. YS: Methodology, Writing—review and editing. GZ: Methodology, Writing—review and editing. LG: Methodology, Writing—review and editing.

## Footnotes

1
http://tree.bio.ed.ac.uk/software/figtree/
